# *APOE* ɛ4 exacerbates age-dependent deficits in cortical microstructure

**DOI:** 10.1093/braincomms/fcad351

**Published:** 2024-02-21

**Authors:** Elijah Mak, Maria-Eleni Dounavi, Grégory Operto, Elina T Ziukelis, Peter Simon Jones, Audrey Low, Peter Swann, Coco Newton, Graciela Muniz Terrera, Paresh Malhotra, Ivan Koychev, Carles Falcon, Clare Mackay, Brian Lawlor, Lorina Naci, Katie Wells, Craig Ritchie, Karen Ritchie, Li Su, Juan Domingo Gispert, John T O’Brien, PREVENT-Dementia and ALFA studies, Katie Bridgeman, Leonidas Chouliaras, Siobhan Coleman, Hannah Darwin, David Driscoll, Maria-Elena Dounavi, Robert Dudas, Sarah Gregory, Ivan Koychev, Brian Lawlor, Audrey Low, Elijah Mak, Clare Mackay, Paresh Malhotra, Jean Manson, Graciela Muniz-Terrera, Lorina Naci, T John O’Brien, Richard Oakley, Vanessa Raymont, Craig Ritchie, Karen Ritchie, William Stewart, Li Su, Peter Swann, Tony Thayanandan, B Guy Williams, Ricardo A Aguilar, Annabella B Gorriti, Anna B Serrat, Raffaele Cacciaglia, Lidia C Gispert, Alba C Martinez, Marta D Milan, Carmen D Gomez, Ruth D Iglesias, Marie E F Karine, Sherezade F Julian, Patricia G Serra, Juan D Gispert, Armand G Escalante, Oriol G Rivera, Laura H Penas, Gema H Rodriguez, Jordi H Ninou, Laura I Gamez, Iva Knezevic, Paula M Alvarez, Tania M Diaz, Carolina M Gil, Eva Palacios, Maria Pascual, Albina P Ballester, Sandra P Mendez, Irina A Radoi, Blanca R Fernandez, Laura R Freixedes, Aleix S Vila, Gonzalo A Sanchez Benavides, Mahnaz S Mahnaz, Lluis S Harster, Anna S Prat, Laura S Stankeviciute, Marc S Calvet, Marc V Jaramillo, Natalia V Tejedor

**Affiliations:** Department of Psychiatry, School of Clinical Medicine, University of Cambridge, Cambridge CB2 0QQ, UK; Department of Psychiatry, School of Clinical Medicine, University of Cambridge, Cambridge CB2 0QQ, UK; Barcelonaβeta Brain Research Center, Pasqual Maragall Foundation, Barcelona 08005, Spain; Department of Psychiatry, School of Clinical Medicine, University of Cambridge, Cambridge CB2 0QQ, UK; Department of Clinical Neurosciences, University of Cambridge, Cambridge CB2 0SZ, UK; Department of Psychiatry, School of Clinical Medicine, University of Cambridge, Cambridge CB2 0QQ, UK; Department of Psychiatry, School of Clinical Medicine, University of Cambridge, Cambridge CB2 0QQ, UK; Department of Psychiatry, School of Clinical Medicine, University of Cambridge, Cambridge CB2 0QQ, UK; Centre for Dementia Prevention, University of Edinburgh, Edinburgh EH4 2XU, UK; Department of Brain Sciences, Imperial College, London W12 0NN, UK; Department of Psychiatry, Oxford University, Oxford OX3 7JX, UK; Barcelonaβeta Brain Research Center, Pasqual Maragall Foundation, Barcelona 08005, Spain; IMIM (Hospital del Mar Medical Research Institute), Barcelona 08003, Spain; Centro de Investigación Biomédica en Red de Bioingeniería, Biomateriales y Nanomedicina (CIBER-BBN), Madrid 28029, Spain; Department of Psychiatry, Oxford University, Oxford OX3 7JX, UK; Institute of Neuroscience, Trinity College Dublin, University of Dublin, Dublin D02 PX31, Ireland; Institute of Neuroscience, Trinity College Dublin, University of Dublin, Dublin D02 PX31, Ireland; Centre for Dementia Prevention, University of Edinburgh, Edinburgh EH4 2XU, UK; Centre for Dementia Prevention, University of Edinburgh, Edinburgh EH4 2XU, UK; Institut National de la Santé et de la Recherche Médicale, U1061 Neuropsychiatrie, Montpellier 34093, France; Faculty of Medicine, University of Montpellier, Montpellier 34093, France; Department of Psychiatry, School of Clinical Medicine, University of Cambridge, Cambridge CB2 0QQ, UK; Barcelonaβeta Brain Research Center, Pasqual Maragall Foundation, Barcelona 08005, Spain; IMIM (Hospital del Mar Medical Research Institute), Barcelona 08003, Spain; Centro de Investigación Biomédica en Red de Bioingeniería, Biomateriales y Nanomedicina (CIBER-BBN), Madrid 28029, Spain; Department of Psychiatry, School of Clinical Medicine, University of Cambridge, Cambridge CB2 0QQ, UK

**Keywords:** NODDI, neurodegeneration, cognitive impairment, preclinical dementia

## Abstract

The apolipoprotein E ɛ4 allele is the primary genetic risk factor for the sporadic type of Alzheimer’s disease. However, the mechanisms by which apolipoprotein E ɛ4 are associated with neurodegeneration are still poorly understood. We applied the Neurite Orientation Dispersion Model to characterize the effects of apolipoprotein ɛ4 and its interactions with age and education on cortical microstructure in cognitively normal individuals. Data from 1954 participants were included from the PREVENT-Dementia and ALFA (ALzheimer and FAmilies) studies (mean age = 57, 1197 non-carriers and 757 apolipoprotein E ɛ4 carriers). Structural MRI datasets were processed with FreeSurfer v7.2. The Microstructure Diffusion Toolbox was used to derive Orientation Dispersion Index maps from diffusion MRI datasets. Primary analyses were focused on (i) the main effects of apolipoprotein E ɛ4, and (ii) the interactions of apolipoprotein E ɛ4 with age and education on lobar and vertex-wise Orientation Dispersion Index and implemented using Permutation Analysis of Linear Models. There were apolipoprotein E ɛ4 × age interactions in the temporo-parietal and frontal lobes, indicating steeper age-dependent Orientation Dispersion Index changes in apolipoprotein E ɛ4 carriers. Steeper age-related Orientation Dispersion Index declines were observed among apolipoprotein E ɛ4 carriers with lower years of education. We demonstrated that apolipoprotein E ɛ4 worsened age-related Orientation Dispersion Index decreases in brain regions typically associated with atrophy patterns of Alzheimer’s disease. This finding also suggests that apolipoprotein E ɛ4 may hasten the onset age of dementia by accelerating age-dependent reductions in cortical Orientation Dispersion Index.

## Teaser

Mak *et al*. demonstrated a significantly more pronounced age-related decline in cortical Orientation Dispersion Index amongst cognitively normal midlife carriers of apolipoprotein E ɛ4 compared with non-carriers. Apolipoprotein E ɛ4 may hasten the onset age of dementia by accelerating age-dependent deficits of cortical microstructure in Alzheimer’s disease regions. Additional longitudinal studies are needed to verify this hypothesis.

## Graphical Abstract

**Graphical Abstract:**
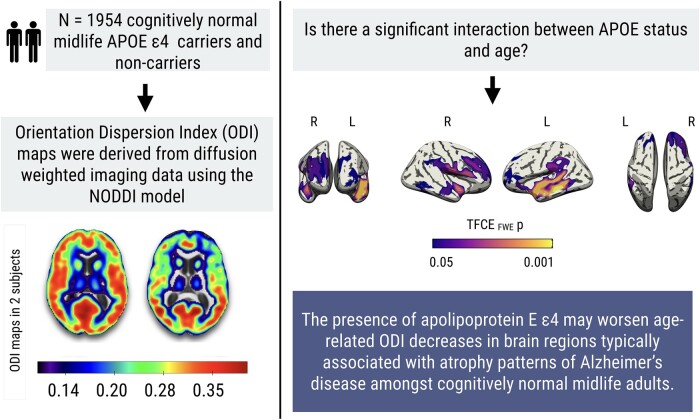

## Introduction

The *APOE* ɛ4 allele is the strongest genetic risk factor for both sporadic early and late-onset Alzheimer’s disease.^1,2^ A growing body of neuroimaging studies has delineated the imaging phenotypes of APOE ɛ4 in cognitively normal individuals as a means to detect preclinical changes that may occur before cognitive impairment (for systematic reviews, see^3,4^) Some notable findings in middle-aged *APOE* ɛ4 carriers include (i) grey matter atrophy in typical Alzheimer’s disease regions,^5-7^ (ii) diffusion tensor imaging (DTI) changes in white matter tracts,^8^ (iii) functional deficits in cerebral perfusion and glucose metabolism^9-11^ as well as (iv) disruptions in both task-based functional MRI^12,13^ and intrinsic connectivity within resting-state networks.^14^

Nonetheless, definitive evidence for a APOE ɛ4 ‘signature’ remains elusive due to null results and inconsistent observations. In addition to methodological discrepancies, small *APOE* ɛ4 effect sizes and sample variability across cohorts, contradictory results may also be attributed to the complex midlife effects of the *APOE* ɛ4 gene as well as its interactions with other pathological markers of Alzheimer’s disease (i.e. amyloid and tau). The associations between *APOE* ɛ4 and brain imaging changes and cognition in midlife adults may to some extent be modulated by sex and cognitive reserve.^15,16^ Similarly, a growing body of evidence suggests that the presence of APOE ɛ4 may exacerbate the trajectory of age-related biomarker changes, with older *APOE* ɛ4 homozygotes exhibiting an accelerated decline in white matter microstructural integrity,^8^ myelination (i.e. MRI T_1_-weighted/T_2_-weighted ratio),^17^ grey matter volumes and hippocampus shape.^18,19^

It is also conceivable that the deleterious effects of *APOE* ɛ4 are more pronounced at the cellular level of neurite morphology (i.e. dendritic arborization) and so would not be easily detected by macroscale analysis of standard MRI biomarkers. This notion is reinforced by histological evidence linking *APOE* ɛ4 to several synaptic and neuritic abnormalities.^20^ Thus, to the extent that neuritic abnormalities precede major cell death or brain atrophy, imaging markers of grey matter microstructure may reveal some of the earliest effects related to *APOE* ɛ4 genotype. To this end, the mean diffusivity (MD) parameter from DTI has been shown to be sensitive to dendritic alterations in the hippocampus,^21^ and its applications have expanded to include the analysis of hippocampal subfields and cortical grey matter in patients with autosomal dominant Alzheimer disease,^22^ mild cognitive impairment (MCI) and Alzheimer’s disease.^23-25^ Collectively, these findings underscore the potential for diffusion MRI to characterize subtle cortical microstructural deficits. However, given the inherent differences in image resolution between typical DTI (2 mm) and T_1_-MRI sequences (1 mm), the cortical MD signal could be sensitive to overestimation due to the spill-in of high MD values from unrestricted ‘free water’ diffusion in the CSF.^26^

Recent advances in diffusion-weighted imaging have led to the development of biophysical models, expanding the application of diffusion imaging beyond white matter to grey matter microstructure.^27^ One particularly promising technique for the quantification of neurite morphology at the level of axons and dendrites is neurite orientation dispersion and density imaging (NODDI), which features a three-compartment model to distinguish signal from intra-neurite, extra-neurite and CSF. The Orientation Dispersion Index (ODI) from NODDI reflects the complexity or dispersion in brain tissue. Histological correlations between ODI and post-mortem measures of orientation complexity suggest that these NODDI and ODI may be used to characterize *in vivo* dendritic branching.^28,29^ Previous studies have also demonstrated strong negative associations between age and ODI, in keeping with the expectation that dendritic arborization reduces with normal ageing.^30,31^ A recent study by our group demonstrated a close relationship between ODI and synaptic density in individuals with primary tauopathies, lending credence to the interpretation of ODI as a proxy of synaptic function.^32^

Given that Alzheimer’s disease pathology accumulates for years before irreversible and observable macrostructural atrophy,^33^ establishing NODDI as a measure of microstructural abnormalities during the asymptomatic period will be essential for early identification, improving prognosis, accurate disease-staging, and treatment monitoring. Though NODDI has shown promise in studies of normal ageing,^8,30^ early-onset Alzheimer’s disease,^34^ and other tauopathies,^32^ there is currently insufficient data to draw conclusions about the influence of APOE ɛ4 on grey matter NODDI measurements in cognitively normal midlife adults. In this multi-site study, the NODDI model is applied to characterize the effects of *APOE* ɛ4 and its interactions with age and education on cortical microstructural changes in cognitively normal individuals recruited from the PREVENT-Dementia^35^ and ALFAprojects.^36^ Both PREVENT-Dementia and ALFA are prospective observational cohort studies with the overarching goal of clarifying the pathophysiology and pathogenic factors occurring during the preclinical phase of Alzheimer’s disease. The goals of this research were to (i) compare *APOE* ɛ4 carriers and non-carriers on measures of ODI measured within the lobes and vertex-wise, (ii) evaluate the extent to which *APOE* ɛ4 interacts with age and education to determine ODI, and (iii) investigate whether NODDI provides novel insights beyond that provided by conventional biomarkers such as cortical thickness.

## Materials and methods

### Study participants

The recruitment for ALFA subjects consisted of two steps. First, 2743 cognitively healthy volunteers aged between 45 and 76 years were enrolled in the ALFA. Exclusion criteria included performance exceeding the established cut-off for a number of cognitive tests and the presence of a psychiatric diagnosis. Second, after *APOE* ɛ4 genotyping, all participants homozygous for the ɛ4 allele as well as carriers of the ɛ2 allele were invited to undergo MRI scanning along with *APOE* ɛ4 heterozygotes and non-carriers, matched for age and sex. The ALFA+ study was approved by the Independent Ethics Committee ‘Parc de Salut Mar’, Barcelona. All participants signed the informed consent form that had also been approved by the Independent Ethics Committee ‘Parc de Salut Mar’, Barcelona; 701 participants were recruited in the PREVENT-Dementia study from 5 study sites: West London (*n* = 210), Edinburgh (*n* = 223), Cambridge (*n* = 100), Oxford (*n* = 68) and Dublin (*n* = 100). The main entry criteria were age 40–59 and the absence of dementia or other neurological disorders. Approval for the study has been given by the NHS Research Ethics Committee London Camberwell St-Giles.

### Neuroimaging

For ALFA, MRI scans were obtained with a 3T scanner (Ingenia CX, Philips, Amsterdam, Netherlands) and included a T_1_-weighted Turbo Field Echo sequence (repetition time = 10 ms, echo time = 5 s, flip angle = 8 and voxel size = 0.75 mm^3^ isotropic) and a diffusion weighted imaging (DWI) protocol (repetition time = 9 ms, echo time = 90 ms, 8 *b*0 volumes, 64 *b* = 1000 s/mm^2^ volumes and voxel size = 2.2 mm^3^ isotropic). For PREVENT, a T_1_-weighted magnetization prepared rapid gradient echo scan was acquired (repetition time = 2.3 s, echo time = 2.98 ms, flip angle = 9, and voxel size = 1 mm^3^ isotropic) on 3T Siemens scanners: Prisma fit (Oxford), Prisma fit (Cambridge), Verio (West London) and Skyra (Dublin and Edinburgh). The DWI protocol in PREVENT was as follows: repetition time = 11 s 700 ms, echo time = 90 ms, 1 *b*0 volumes, 64 *b* = 1000 s/mm^2^ volumes, flip angle = 90, and voxel size = 2 mm^3^ isotropic.

### Preprocessing

Image preprocessing and vertex-wise analyses were performed on a dedicated cluster composed of CPU nodes [Cambridge Service for Data-Driven Discovery (CSD3): 2×Intel Xeon Skylake 6142 processors, 2.6 GHz 16-core, 192GiB RAM per node], and GPU servers for the GPU-optimized parts of the processing pipeline (NVidia Tesla K40/K80 GPUs).

### Structural MRI

T_1_ structural MRI datasets from ALFA and PREVENT were preprocessed using identical procedures. MRI scans were first corrected for intensity inhomogeneities with the Advanced Normalization Toolbox N4 algorithm.^37^ Automated surface-based reconstruction of the T_1_-MRI datasets was performed using FreeSurfer v7.2 (http://surfer.nmr.mgh.harvard.edu/).^38^ Visual inspections of the scans were performed by two authors (E.M. and E.T.Z) to ensure that there were no severe errors in the surface constructions (i.e. global misclassification of grey and white matters). Minor edits were made conservatively on datasets with white matter or pial surface errors when necessary (see Supplementary Materials for an example of topological correction). Estimates of cortical thickness were determined using Freesurfer by computing the average distance between grey matter/white matter and grey matter/CSF surfaces at each vertex across the cortical mantle. These thickness measurements were then projected to the inflated surface of the rebuilt brain of each subject.

### Diffusion MRI

#### Preprocessing

Diffusion datasets were visually inspected for optimal coverage and to ensure minimal eddy-current distortions. Subjects were excluded if there were excessive Echo Planar Imaging (EPI) distortions, poor image quality or insufficient field-of-view coverage. First, the *dwidenoise* tool from MRTRIX (https://www.mrtrix.org) was used to perform denoising on the raw 4D DWI volumes to improve signal-to-noise ratio.^39^ Second, the *mrdegibbs* tool was used to apply Gibbs ringing correction to get rid of ringing artefacts. Subsequently, the DWI datasets were stripped of nonbrain tissue using the Brain Extraction Tool. To confirm the accuracy of the masks, a visual inspection was undertaken, and manual modifications were performed as needed. Eddy currents and head movements were corrected with *eddy_openmp* in FSL (Version 6.0.5). Quantitative identification of slices with signal loss was performed using the *-repol* option, and outlier volumes were replaced by non-parametric predictions using the Gaussian process.^40^ The *b*-matrix was subsequently reoriented by applying the rotational part of the affine transformation used during eddy correction.^41^

#### Neurite orientation dispersion and density imaging

Following eddy-current correction, we applied the NODDI model^27^ using the Microstructure Diffusion Toolbox^42^ to generate ODI whole-brain maps. The diffusion signal within each brain voxel is described by NODDI as a combination of intracellular, extracellular and CSF components. The intracellular compartment is modelled as a set of sticks with restricted diffusion perpendicular to the orientation of the axonal bundles and unimpeded diffusion along them in order to capture neurite membranes and myelin sheaths. The extracellular compartment is assumed to encompass the region surrounding the neurites, which is made up of glial cells and somas. Isotropic diffusion is used to represent the CSF compartment. The ODI is a tortuosity measure computed as the neurite dispersion coefficient. Values closer to 0 indicate less dispersion or well-aligned neurites, but values closer to 1 indicate greater dispersion, which is often associated with increased dendritic branching or complexity in grey matter.^29^ The original paper established the feasibility of modelling the ODI from single-shell diffusion measurements.^27^ Furthermore, single-shell ODI studies have been demonstrated to reproduce group-level differences from multi-shell DWI data, particularly analyses were limited to the grey matter.^43^ The neurite density (NDI) maps were excluded from all analyses since prior research found significant discrepancies between the NDI maps from multi- and single-shell DWIs.^27,43^

#### Coregistration and estimation of lobar ODI

The *b*0 volumes were coregistered with the T_1_-MRI using Freesurfer’s Boundary-Based Registration.^44^ In order to warp the cortical lobar labels to native DWI space using the inverse transformation that was utilized to coregister the *b*0 to the T_1_-MRI, we used the mri annotation2label command with the ‘-lobesStrict’ flag, followed by mri label2vol and mri vol2vol. The average ODI values for the parietal, occipital, temporal, cingulate and insula lobes were generated using ‘mri segstats’. Given the inherent resolution differences between DWI and T_1_-MRI, the lobar methodology depends less on the perfect alignment of fine sulcal areas and provides a robust method for detecting *APOE* ɛ4-related alterations in the presence of potential image-coregistration difficulties Furthermore, it has been shown that lobe averaged values of imaging derived cortical thickness levels were comparable to the range of values as reported by von Economo’s histological measurements.^45,46^

#### Vertex-wise ODI surfaces

In addition, we performed a vertex-wise analysis to provide a more nuanced understanding of the correlations between *APOE* ɛ4 and topological ODI changes. In order to accomplish this, the ODI intensities were sampled from the midpoint of the cortical ribbon (i.e. 50% of the cortical thickness along the surface normal to the grey matter/white matter surface) and projected to an inflated surface using the mri vol2surf function in Freesurfer, before smoothing at 15-mm Guassian kernel.

#### ComBat harmonization

To attenuate site-related effects of ODI, we used ComBat to lobar ODI and vertex-wise ODI datasets using R and custom MATLAB scripts, respectively.^47^ Age, sex and *APOE* ɛ4 status were specified as biological factors to retain the biological variability of the data, as demonstrated by the expected correlations with age (Supplementary Materials). The outcomes of the multi-site harmonization and the effects of ComBat on ODI are depicted in subject-specific box plots of lobar ODI, ordered by the site (Supplementary Materials). After using ComBat, site effects on ODI were no longer statistically significant.

#### Statistical analyses

All statistical analyses were performed in R version 4.2.0 (22 April 2022). Demographic variables and characteristics of the cohort were reported as mean (standard deviation, SD) or number (percentage), and differences between the *APOE* ɛ4 carriers and non-carriers were compared using ANOVA, Kruskal–Wallis, and *χ*^2^ tests, where appropriate. Individuals with the ɛ2/ɛ4, ɛ3/ɛ4 and ɛ4/ɛ4 genotypes were classified as *APOE* ɛ4 carriers and compared against the non-carriers (ɛ2/ɛ2, ɛ2/ɛ3 and ɛ3/ɛ3 genotypes). Our primary analyses focused on the (i) main effects of *APOE* ɛ4, and the (ii) interactions of *APOE* ɛ4 with age on lobar and vertex-wise ODI. Non-parametric permutation models were computed using Permutation Analysis of Linear Models (PALM; https://fsl.fmrib.ox.ac.uk/fsl/fslwiki/PALM). In addition, the quadratic term of age was examined in order to model nonlinear changes. Thus, a significant main effect of *APOE* ɛ4 would imply group-level differences in ODI, whereas a significant interaction term would be consistent with a ‘accelerating’ effect of *APOE* ɛ4 on age-dependent ODI changes. The R package ‘emmeans’ was used to estimate marginal effects and to estimate the earliest age at which differences between *APOE* ɛ4 carriers and non-carriers could emerge. (iii) Non-parametric permutation models were used to assess three-way interactions between formal education years, age, and *APOE* ɛ4 on ODI to determine the potential role of cognitive reserve in moderating the effects of *APOE* ɛ4 on ODI changes. Statistical results were reported after correcting for multiple comparisons using family wise error (FWE). The TFCE method was used to adjust for multiple comparisons in all vertex-wise analyses. Vertex-wise and lobar ODI results were visualized using Surfice (https://www.nitrc.org/plugins/mwiki/index.php/surfice:MainPage) and the R package GGSEG, respectively.

## Results

### Sample characteristics

Participant characteristics are summarized in Table 1. Across the total sample (*N* = 1954), there were 1197 non-carriers [ɛ2/ɛ2: *N* = 5 (0.3%), ɛ2/ɛ3: *N* = 133 (6.8%), ɛ3/ɛ3: *N* = 1059 (54.2%)], and 757 *APOE* ɛ4 carriers [ɛ2/ɛ4: *N* = 43 (2.2%), ɛ3/ɛ4: *N* = 616 (31.5%) and ɛ4/ɛ4: *N* = 98(5%)]. The mean (SD) age of the sample was 57 ± 7.3, ranging from 40 to 77.1. There were no significant differences in age (*P* = 0.73), sex distribution (*P* = 0.44) and years of education (*P* = 0.23) between the *APOE* ɛ4 carriers and non-carriers.

**Table 1:** Sample characteristics of the study sample

Variable	Overall, *N* = 1954	ɛ4+, *N* = 757	ɛ4−, *N* = 1197	*P*-value^a^
Age				0.73
Mean (SD)	57.03 (7.26)	56.92 (7.31)	57.10 (7.23)	
Range	40.00, 77.08	40.00, 75.36	40.00, 77.08	
Sex, *n* (%)				0.44
Female	1221 (62%)	465 (61%)	756 (63%)	
Male	733 (38%)	292 (39%)	441 (37%)	
Education (years)				0.23
Mean (SD)	14.43 (3.78)	14.58 (3.86)	14.34 (3.73)	
Range	0.00, 38.00	6.00, 38.00	0.00, 27.00	

^a^Wilcoxon rank sum test; Pearson's Chi-squared test.

Abbreviation: *APOE*, Apolipoprotein E gene.

### Main effects of *APOE* ɛ4

**Figure 1:**
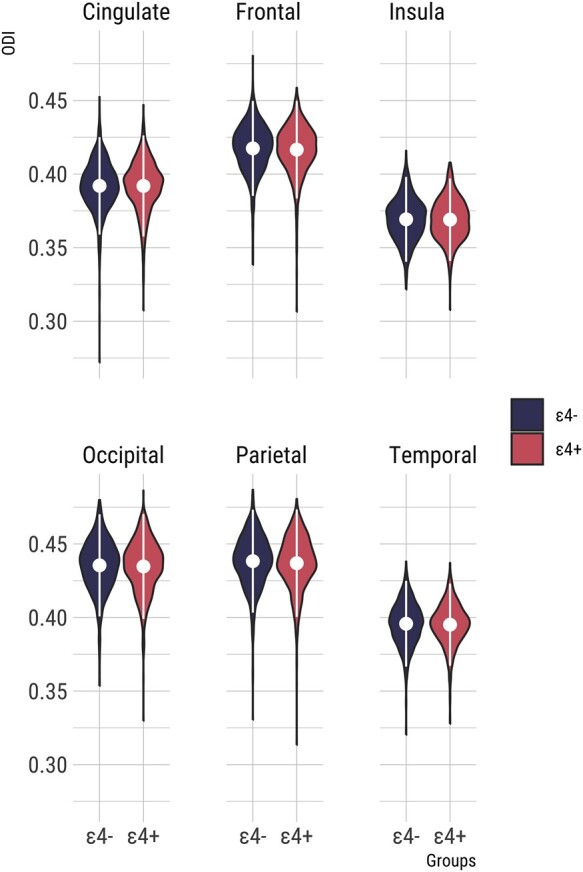
**Violin plots of mean lobar ODI in *APOE* ɛ4 carriers and non-carriers.** Non-parametric permutation tests of group differences using PALM revealed no significant differences between both groups after adjusting for multiple comparisons with FWE (frontal: *T* = 1.5, *P*_FWE_ = 0.82, parietal: *T* = 2.2, *P*_FWE_ = 0.75, temporal: *T* = 0.9, *P*_FWE_ = 0.88, occipital: *T* = 1.1, *P*_FWE_ = 0.86, cingulate: *T* = 0.2, *P*_FWE_ = 0.98, insula: *T* = 0.4, *P*_FWE_ = 0.94). Abbreviations: *APOE*, Apolipoprotein E gene; FWE, family wise error; ODI, Orientation Dispersion Index; PALM, Permutation Analysis of Linear Models.

Violin plots of lobar ODI in *APOE* ɛ4 carriers and non-carriers are shown in Fig. 1. Both groups did not differ in terms of lobar ODI (frontal: *T* = 1.5, *P*_FWE_ = 0.82, parietal: *T* = 2.2, *P*_FWE_ = 0.75, temporal: *T* = 0.9, *P*_FWE_ = 0.88, occipital: *T* = 1.1, *P*_FWE_ = 0.86, cingulate: *T* = 0.2, *P*_FWE_ = 0.98, insula: *T* = 0.4, *P*_FWE_ = 0.94), and effect sizes were small (Cohen’s *D*: 0.01–0.13). Similarly, vertex-wise analyses revealed no significant clusters of ODI differences between the two groups (threshold free cluster enhancement with FWE rate adjustment; TFCE *P*_FWE_ > 0.05).

### *APOE* ɛ4 × age

There were significant *APOE* ɛ4 × age interactions in the frontal, parietal, temporal, cingulate and insula lobes, indicating differential age-dependent ODI reductions in *APOE* ɛ4 carriers relative to non-carriers (frontal: *T* = 2.7, parietal: *T* = 2.7, temporal: *T* = 3.6, cingulate: *T* = 2.1, insula: *T* = 3, *P*_FWE_ < 0.05).

**Figure 2:**
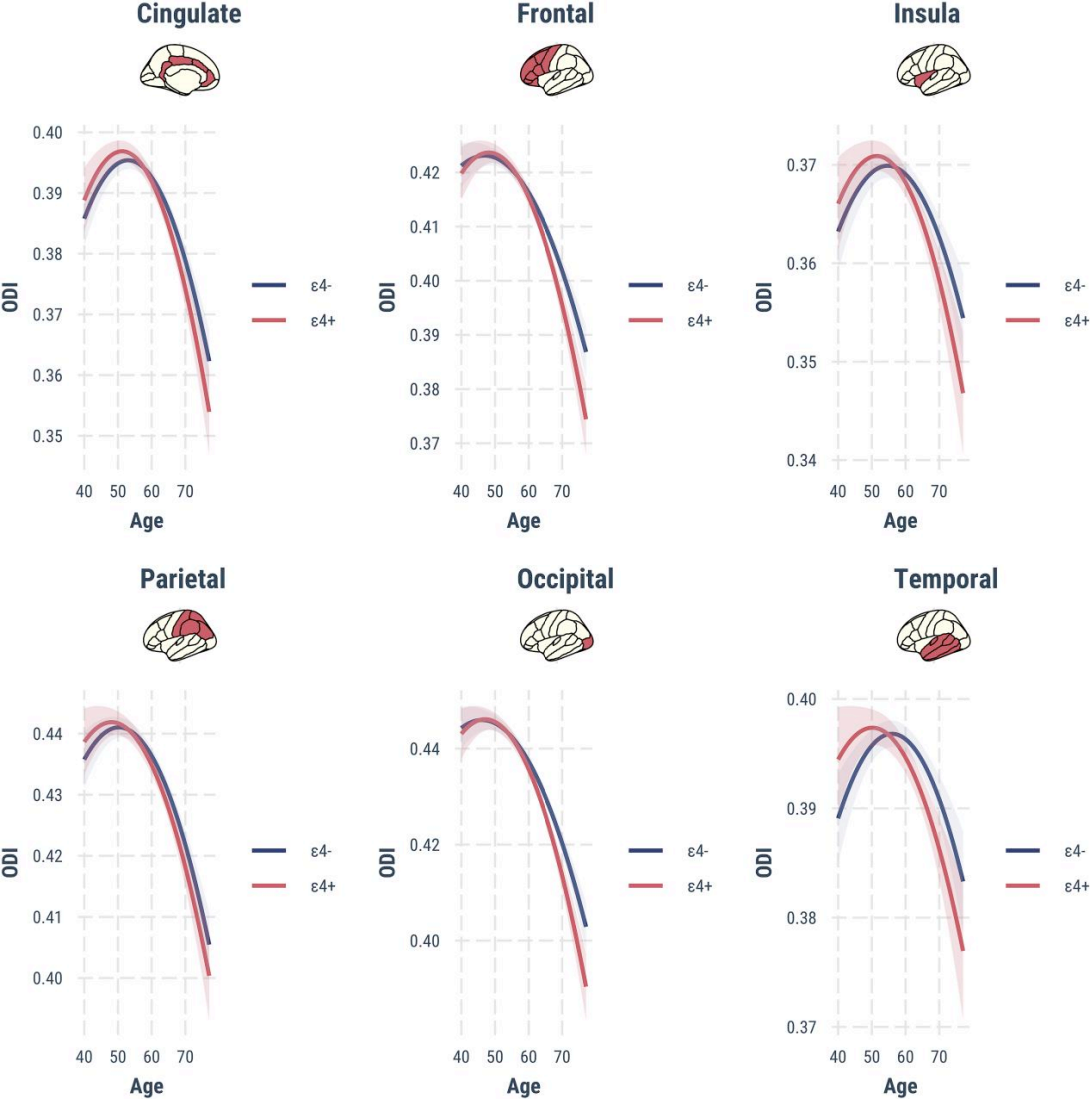
**Marginal effect graphs depicting the two-way interactions between *APOE* ɛ4 and age (*x*-axis) on lobar ODI values (*y*-axis).** Individuals with the APOE ɛ4 allele showed significantly steeper age-related reductions in ODI compared with non-carriers in the cingulate, frontal, insula, parietal and temporal lobes. Statistical significance was determined in PALM using non-parametric permutation models (5000 permutations, *P*_FWE_ < 0.05, adjusted for gender, years of formal education and sites). *APOE*, Apolipoprotein E gene; FWE, family wise error; ODI, Orientation Dispersion Index; PALM, Permutation Analysis of Linear Models.

**Figure 3:**
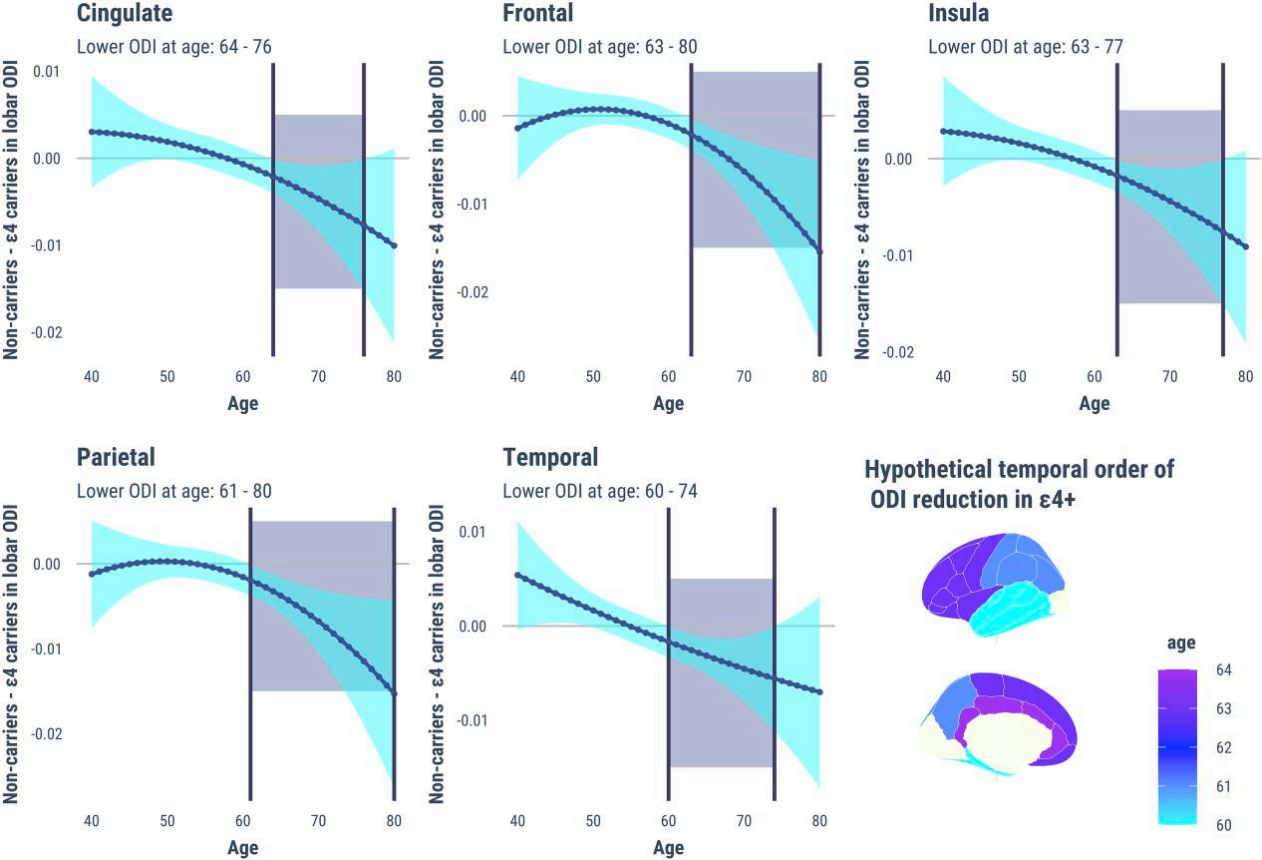
**Differences in standardized lobar ODI between *APOE* ɛ4 carriers and non-carriers (*y*-axis) as a function of age (*x*-axis).** Pairwise comparisons were calculated based on marginal effects to predict the earliest hypothetical age at which *APOE* ɛ4 carriers would show decreased lobar ODI relative to non-carriers, highlighted by the vertical dotted lines on the *x*-axis. The shaded cyan region represents the 95% confidence interval. For each lobe, the earliest age of significant ODI reductions in *APOE* ɛ4 carriers is illustrated in the choropleth brain map. Abbreviations: *APOE*, Apolipoprotein E gene; ODI, Orientation Dispersion Index.

Plots of marginal effects showed steeper age-related decreases in ODI for *APOE* ɛ4 carriers compared with non-carriers Fig. 2. To estimate the hypothetical inflection points of divergent ODI age trends, we calculated between-group differences of the predicted ODI from age 40 to 80 and determined the earliest age at which the differences emerged (Fig. 3). In general, lobar ODIs were similar between *APOE* ɛ4 carriers and non-carriers from age 40 to 60, after which significant reductions in ODI became more apparent in *APOE* ɛ4 compared with the non-carriers (temporal: age = 60–74, cingulate: age = 64–76, frontal: age = 63–80, insula: age = 63–77 and parietal: age = 61–80).

**Figure 4:**
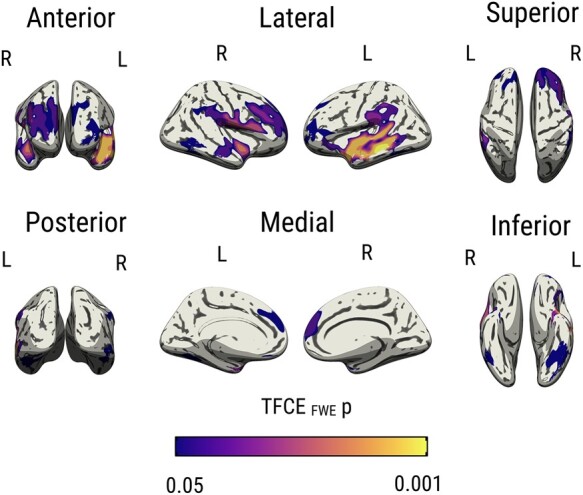
**Vertex-wise interactions between *APOE* ɛ4 and age on cortical ODI.** Results were obtained using non-parametric permutation models, implemented using PALM on ComBat harmonized ODI surface maps, and significance was established with TFCE *P*_FWE_ < 0.05, adjusted for sex, years of formal education and site. Abbreviations: *APOE*, Apolipoprotein E gene; TFCE, Threshold Free Cluster Enhancement; FWE, Family Wise Error; ODI, Orientation Dispersion Index; PALM, Permutation Analysis of Linear Models.

**Figure 5:**
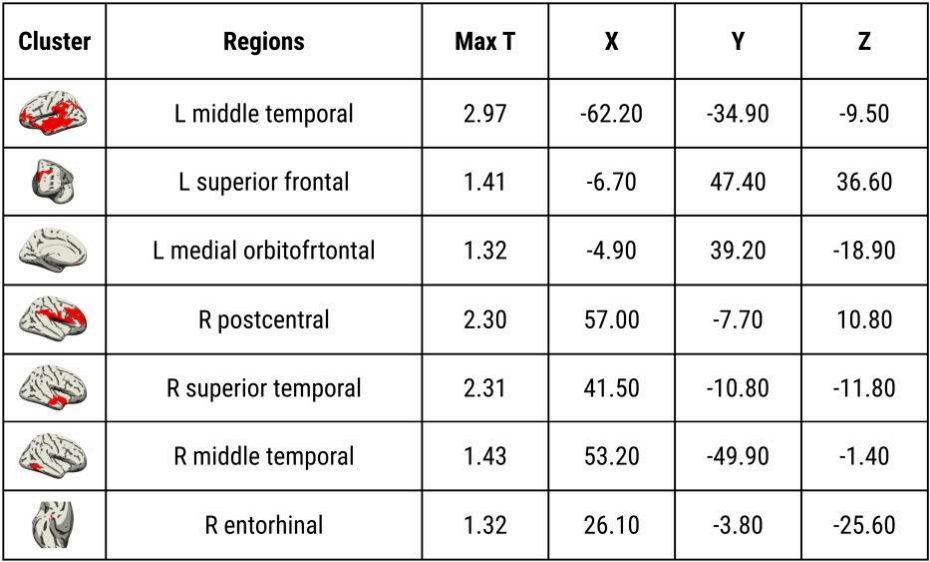
**Summary statistics and MNI152 coordinates significant cluster associated with the *APOE* ɛ4×age interaction on vertex-wise ODI (TFCE *P*_FWE_ < 0.05, adjusted for sex, years of formal education, and site).** Abbreviations: *APOE*, Apolipoprotein E gene; TFCE, Threshold Free Cluster Enhancement; FWE, family wise error; ODI, Orientation Dispersion Index; MNI152, Montreal Neurological Institute 152.

Next, we used an unbiased vertex-wise approach to map the topography of the *APOE* ɛ4 age interactions across the cortex. Significant clusters were found primarily in the temporo-parietal cortices and the frontal lobe (Fig. 4). Figure 5 tabulates the summary statistics and MNI coordinates of the peak effects in each cluster. There were no significant interactions between *APOE* ɛ4 and age on lobar cortical thickness (*P*_FWE_ > 0.05).

### *APOE* ɛ4 × age × education interactions

**Figure 6:**
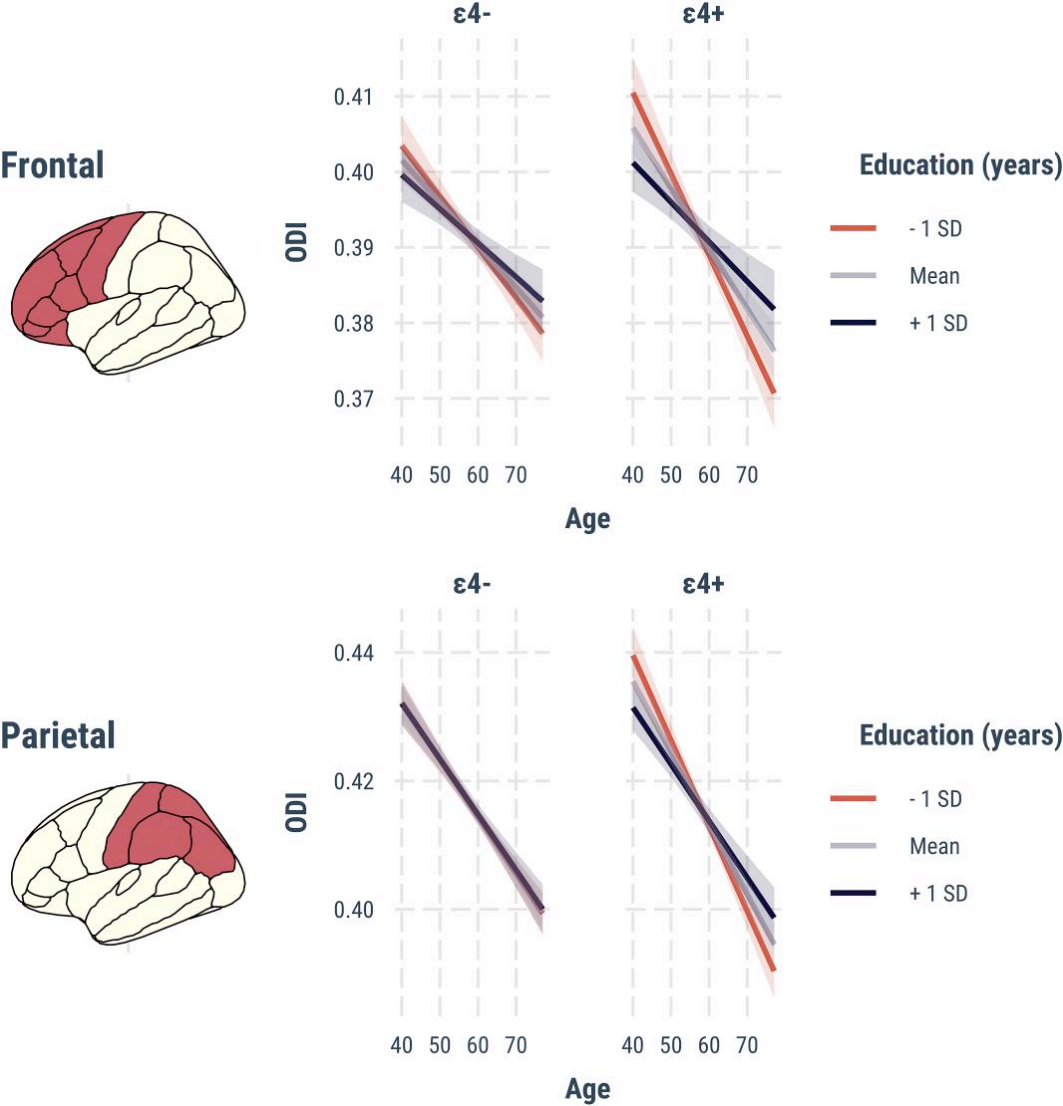
**Three-way interactions between *APOE* ɛ4 × age × education.** Marginal effects are depicted for the frontal and parietal lobes, stratified by *APOE* ɛ4 status. Lower years of education (mean–1 SD) are associated with steeper age-dependent reductions of ODI among the ɛ4 carriers only (frontal: *T* = 2.68, parietal: *T* = 2.37, *P*_FWE_ < 0.05, adjusted for site and sex). Abbreviations: *APOE*, Apolipoprotein E gene; FWE, family wise error; SD, standard deviation; ODI, Orientation Dispersion Index.

Three-way interactions of *APOE* ɛ4 × age × education were found in the frontal, parietal lobes (*P*_FWE_ < 0.05), indicating steeper age-related reductions in ODI among *APOE* ɛ4 carriers with lower years of education (mean–1 SD). *Post hoc* stratified analyses in *APOE* ɛ4 carriers and non-carriers confirmed that the age × education interaction was significant only among *APOE* ɛ4 carriers [frontal: *T* = 2.68, parietal *T* = 2.37 and *P*_FWE_ < 0.05 (Fig. 6)].

**Figure 7:**
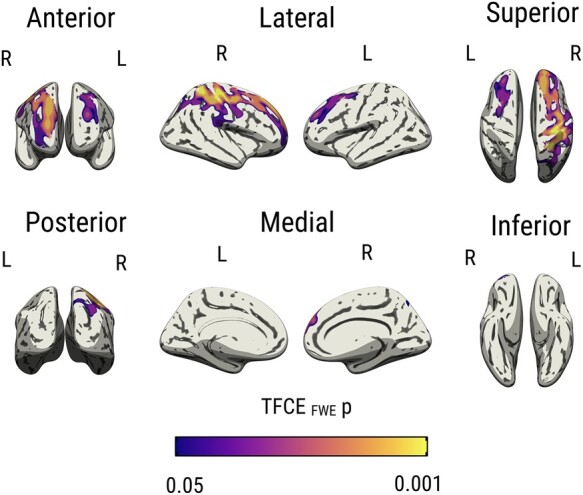
**Vertex-wise three-way interactions between *APOE* ɛ4 × age × education.** Marginal effects are depicted for the frontal and parietal lobes, stratified by *APOE* ɛ4 status. Lower years of education (mean–1 SD) are associated with steeper age-dependent reductions of ODI among the ɛ4 carriers only (*P*_FWE_ < 0.05, adjusted for site and sex). Abbreviations: *APOE*, Apolipoprotein E gene; FWE, family wise error; SD, standard deviation; ODI, Orientation Dispersion Index.

**Figure 8:**
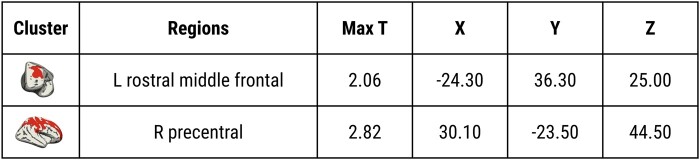
**Summary statistics and MNI152 coordinates for each significant cluster associated with the three-way interactions of *APOE* ɛ4×age × education interaction on vertex-wise ODI.** Abbreviations: *APOE*, Apolipoprotein E gene; ODI, Orientation Dispersion Index; MNI152, Montreal Neurological Institute 152.

The fronto-parietal lobar patterns of *APOE* ɛ4 × age × education interactions were corroborated by vertex-wise permutation analyses, which revealed peak effects in the rostral left middle frontal and precentral gyri (Fig. 7; TFCE *P*_FWE_ < 0.05). The summary statistics and MNI coordinates of the peak effects in each cluster are shown in Fig. 8. There were no significant three-way interactions between *APOE* ɛ4 × age × education on lobar cortical thickness.

### Sensitivity analyses

A series of sensitivity analyses were performed to determine the robustness of our key findings against potential confounds. (i) All analyses were repeated after excluding the *APOE* ɛ2ɛ4 carriers due to the genetic ambiguity of contrasting risk profiles. (ii) To investigate whether the *APOE* ɛ4 effects on ODI are independent of atrophy, we additionally adjusted for mean cortical thickness. All the findings were unchanged. Detailed results for these analyses are provided in the Supplementary Materials.

## Discussion

Our findings demonstrate more pronounced age-related declines in cortical ODI among APOE ɛ4 carriers compared with non-carriers. The interactions of *APOE* ɛ4 and age were most prominent in the temporo-parietal lobes, which are known to be more vulnerable to early neurodegeneration and accumulation of Alzheimer’s disease abnormalities. Second, our analyses of the three-way interactions between *APOE* ɛ4, age, and education showed that the detrimental effects of *APOE* ɛ4 with increased age were most strongly expressed in ɛ4 carriers with lower levels of education, suggesting that high levels of cognitive reserve could mitigate against the accelerated ODI decline in ɛ4 carriers. Finally, these findings were independent of cortical atrophy. While conclusions are limited due to the cross-sectional design, our findings imply that *APOE* ɛ4-related changes in ODI may precede macroscopically visible changes in cortical atrophy and may be a more sensitive marker of incipient Alzheimer’s disease.

A growing body of evidence suggests that *APOE* ɛ4 is a potent moderator of age-related trends for imaging biomarkers.^17,19,48,49^ Indeed, we observed widespread interactions between age and *APOE* ɛ4 on ODI, characterized by steeper age-dependent ODI reductions in *APOE* ɛ4 carriers compared with non-carriers. Insofar as ODI is a valid proxy for dendritic complexity,^28,29^ our findings could also be interpreted as an *in vivo* extension of a previous histological study in which Alzheimer’s disease *APOE* ɛ4 carriers exhibited a marked loss of dendritic outgrowth and synaptic count relative to non-carriers.^50^ Loss of dendritic arborization is a common phenomenon associated with the ageing brain,^51^ and previous NODDI studies have demonstrated age-related decline with ODI.^30,52^ The main novel aspect of our finding, however, is that the presence *APOE* e4 allele may further exacerbate the age-dependent decline in ODI. The interactions between *APOE* e4 and age are also broadly consistent with data from other imaging modalities, including accelerated grey matter atrophy,^53^ alterations in myelination,^17^ hippocampal shape,^18,19^ glucose metabolism^54^ and cerebral perfusion.^10,55^ More broadly, these studies suggest that the previously outlined inconsistencies in the literature could be clarified by taking into account the non-uniform effects of APOE4 across age in the future.

Marginal effect analyses revealed that cortical ODI trajectories diverged between *APOE* 4 allele carriers and non-carriers around the age of 60 (i.e. temporal lobe). While additional longitudinal studies are required to validate these results, our data may hint at the existence of an inflection point (age = 60) prior to the appearance of more severe grey matter deficits in *APOE* ɛ4 carriers. Multiple lines of research seem to support this notion. For instance, it has been shown that *APOE* ɛ4 is more closely associated with Alzheimer’s disease neuropathology after the age of 60.^56^ Within the context of current biomarker models in Alzheimer’s disease,^57^ our results positioned the onset of *APOE* ɛ4-related ODI alterations (age = 60) in close temporal proximity with the exponential build-up of amyloid (age = 60),^58^ roughly a decade before the onset of cognitive impairment *APOE* ɛ4 (age = 69).^59,60^ These studies, including our own findings, lend support to the hypothesis that the detrimental consequences of *APOE* ɛ4 are more pronounced after the sixth decade.

Although the molecular pathways by which age and *APOE* ɛ4 interact to influence cortical microstructure remain poorly understood, several lines of research support the biological plausibility of our findings. (i) First, *APOE* ɛ4 may influence age-related ODI declines via tau-related pathways.^58,61^ Recent evidence suggests that cognitively normal *APOE* 4 carriers accumulate tau and amyloid at a quicker rate beginning at age 60, which coincides with our findings in the temporal lobe ODI (earliest divergence between *APOE* 4 and non-carriers at age 60).^58^ Indeed, our vertex-wise analyses demonstrated that the most prominent effects were localized within the temporal cortices (e.g. entorhinal cortex, inferior temporal cortex)—early predilection sites for neurofibrillary tangles.^62^ Previous data from transgenic mice^63^ and neuropathological studies have also shown that *APOE* ɛ4 is related to increased tau deposition in the temporal cortex.^64^ In addition, lower cortical ODI has been observed in rTg4510 transgenic mice model.^65^ Our group has recently demonstrated *in vivo* reductions of ODI in patients with primary taupathies,^32^ and lower ODI has been correlated with increased uptake of the PET [18F]THK5351 tracer in an Alzheimer’s disease sample.^66^ (ii) Second, amyloid deposition may also play a role, since 10–15% of *APOE* ɛ4 participants in our cohorts are anticipated to have elevated amyloid burden.^67,68^ It has been shown that dendritic branching in close proximity to amyloid deposits exhibits bending abnormalities, more abrupt branch terminations and spine loss.^69^ Future research integrating multi-modal imaging with CSF or plasma biomarkers^70^ would be well-suited to distinguish the unique contributions of Alzheimer’s disease pathologies to changes in cortical ODI. In order to establish whether our vertex-wise findings reflect the ‘penumbra’ of subsequent atrophy, longitudinal studies comprising a wider spectrum of cognitively normal *APOE* ɛ4 carriers, individuals with MCI and Alzheimer’s disease are warranted.

A secondary aim of this study was to determine the degree to which education may attenuate the adverse effects of *APOE* ɛ4 on age-related ODI changes. To that end, our lobar and vertex-wise analyses indicated a significant three-way interaction between age, *APOE* ɛ4 and education years in the fronto-parietal cortices; i.e. steeper age-dependent ODI reductions were observed in *APOE* ɛ4 carriers with less education. In contrast, the impact of *APOE* ɛ4 on age-related ODI changes in individuals with more years of education was negligible. As such, our findings lend support to previous findings indicating that more years of education was a significant predictor of less cognitive change over time among ApoE e4 carriers^71^ and are aligned with post-mortem evidence, suggesting that higher education may mitigate the deleterious effects of senile plaques^72^ The involvement of fronto-parietal cortices may also be interpreted in light of their roles as key neural substrates of working memory.^73^ Indeed, a recent study has shown that education beyond high school may mitigate the effects of *APOE* ɛ4 on working memory.^15^ Collectively, these findings support a scenario in which a diminished ‘cognitive reserve’ may lead to greater age-related susceptibility to the *APOE* ɛ4 allele.^16^

Notably, our ODI findings persisted after controlling for cortical thickness. Moreover, we did not find any significant effects of *APOE* ɛ4 carriership on cortical thickness (i.e. effects may only appear in *APOE* ɛ4 homozygotes at this age range). The null results of *APOE* ɛ4 on cortical thickness could be anticipated in light of the inconsistent findings and modest effect sizes in the literature. Grey matter atrophy is also thought to reflect downstream neurodegenerative changes occurring later in the course of Alzheimer’s disease.^74^ Under the assumption that a cross-sectional estimate of a biomarker represents its accumulated pathological burden, the combination of ODI changes and the absence of cortical thickness effects is compatible with early loss of dendritic arborization before cell death and atrophy.^75,76^ Similarly, greater magnitudes of changes seen in NODDI relative to that of atrophy have been demonstrated in studies of people with primary tauopathies,^32^ young-onset Alzheimer’s disease,^34^ MCI and Alzheimer’s disease.^77^ Taken together, our findings suggest that NODDI may serve as a promising biomarker to reveal unique insights into subtle grey matter deficits in preclinical dementia.

There are several caveats to consider when interpreting our results. (i) Our cross-sectional design precludes definitive conclusions regarding the role of APOE ɛ4 in predicting age-related trends of ODI reduction. Therefore, conclusions of group differences at different ages should be considered preliminary. Prospective longitudinal studies are necessary to determine if the intra-individual rates of ODI reductions are (ii) significantly accelerated in *APOE* ɛ4 carriers and (iii) whether they are associated with cognitive decline. While our findings revealed robust associations between *APOE* ɛ4 and accelerated reductions in ODI, the pathological processes underpinning this relationship remain unclear. Additional investigations using fluid biomarkers or PET imaging would be required to characterize the pathogenic processes that may underlie the *APOE* ɛ4-related ODI alterations. Leveraging multi-modal data will facilitate analyses to investigate potential interactions of *APOE* ɛ4 with other pathological markers on ODI. The lack of amyloid status on our participants is another limitation, and future studies are needed to determine whether findings are consistent in amyloid-negative *APOE* ɛ4 carriers. (iv) The ODI parameters were determined from diffusion-weighted single-shell datasets as opposed to multi-shell NODDI sequences. Nonetheless, the feasibility of estimating ODI from single-shell DWI had been demonstrated in the seminal NODDI study,^27^ and subsequent groups have confirmed that ODI maps calculated using multi-shell and single-shell (*b* = 1000 s/mm^2^) are highly comparable and exhibit a high degree of overlap with respect to group differences.^43^ (v) While we have interpreted ODI reductions as suggestive of dendritic arborization loss, histological validation of NODDI measurements remains confined to post-mortem spinal cord specimens from patients with multiple sceleroides.^29^ More research triangulating data from *ex vivo* imaging, post-mortem samples and PET imaging would be required to draw firmer conclusions on the specific cellular abnormalities revealed by ODI. (vi) Lower education years increased the detrimental effect of *APOE* ɛ4 on age-related ODI declines; however, interpretations should also account for the extensive relationships of education with general health, environmental enrichment and socioeconomic status, all of which could significantly moderate the associations between education and *APOE* ɛ4-related ODI decreases. (vii) Another limitation of the current study is our exclusive focus on older adults with normal cognitive function. By restricting our sample to non-impaired individuals, we aimed to delineate structural brain changes associated with *APOE* ɛ4 in the preclinical stage, prior to the onset of overt symptoms. Therefore, our findings may not generalize to later stages of dementia, where relationships between *APOE* ɛ4 status and brain structure may be more complex.

In summary, we have conducted a comprehensive analysis of *APOE* ɛ4 carriership and its impact on age-related patterns of ODI alterations in one of the largest datasets of cognitively normal persons in midlife. We found novel evidence that *APOE* ɛ4 worsened age-related ODI decreases in brain regions typically associated with atrophy patterns of Alzheimer’s disease. This finding also suggests that *APOE* ɛ4 may hasten the onset age of dementia by accelerating age-dependent reductions in cortical ODI, although additional studies are needed to verify this hypothesis. Finally, our research lends methodological support to the use of NODDI as a sensitive surrogate for changes in grey matter microstructure.

## Supplementary Material

fcad351_Supplementary_Data

## Data Availability

The data supporting the conclusions described in this paper are available upon reasonable request from the corresponding author.

## Appendix

### Members of the PREVENT Dementia group

Katie Bridgeman, Leonidas Chouliaras, Siobhan Coleman, Hannah Darwin, David Driscoll, Maria-Elena Dounavi, Robert Dudas, Sarah Gregory, Ivan Koychev, Brian Lawlor, Audrey Low, Elijah Mak, Clare Mackay, Paresh Malhotra, Jean Manson, Graciela Muniz-Terrera, Lorina Naci, T. John O’Brien, Richard Oakley, Vanessa Raymont, Craig Ritchie, Karen Ritchie, William Stewart, Li Su, Peter Swann, Tony Thayanandan, B. Guy Williams.

### Members of the ALFA study group

Ricardo A. Aguilar, Annabella B. Gorriti, Anna B. Serrat, Raffaele Cacciaglia, Lidia C. Gispert, Alba C. Martinez, Marta D. Milan, Carmen D. Gomez, Ruth D. Iglesias, Marie E. F. Karine, Sherezade F. Julian, Patricia G. Serra, Juan D. Gispert, Armand G. Escalante, Oriol G. Rivera, Laura H. Penas, Gema H. Rodriguez, Jordi H. Ninou, Laura I. Gamez, Iva Knezevic, Paula M. Alvarez, Tania M. Diaz, Carolina M. Gil, Eva Palacios, Maria Pascual, Albina P. Ballester, Sandra P. Mendez, Irina A. Radoi, Blanca R. Fernandez, Laura R. Freixedes, Aleix S. Vila, Gonzalo A. Sanchez Benavides, Mahnaz S. Mahnaz, Lluis S. Harster, Anna S. Prat, Laura S. Stankeviciute, Marc S. Calvet, Marc V. Jaramillo, Natalia V. Tejedor.
