# Supplementary material for: APOE ɛ4 exacerbates age-dependent deficits in cortical microstructure
Source: Brain Commun. 2024 Feb 21;6(1):fcad351. doi: 10.1093/braincomms/fcad351 (PMC10881196; doi:10.1093/braincomms/fcad351)
Supplement: fcad351_Supplementary_Data [file fcad351_supplementary_data.docx]

# SUPPLEMENTARY MATERIALS

#
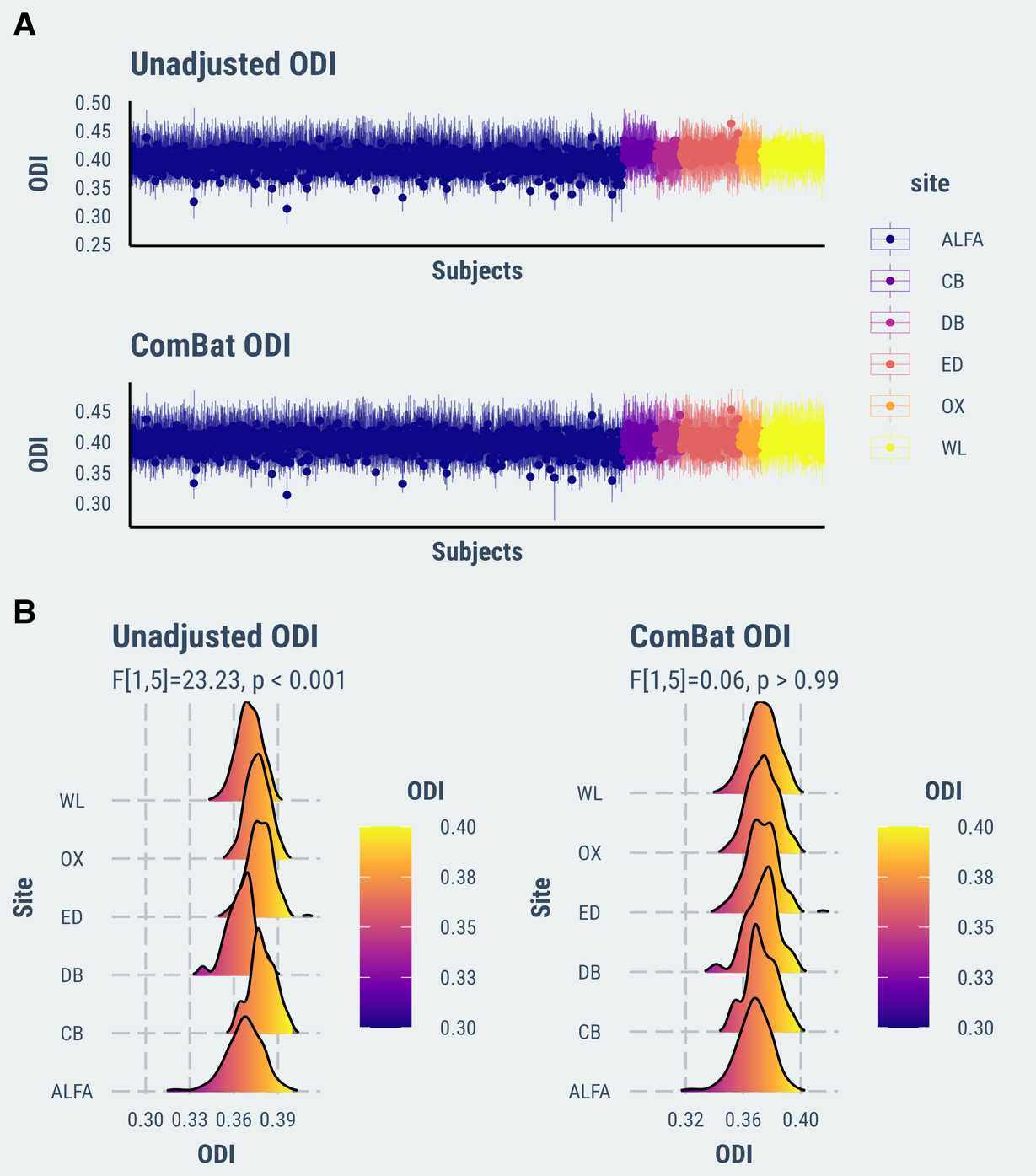


| **Supplementary Fig 1.** ComBat harmonisation of the dataset. A: Each boxplot depicts the lobar ODI values of a subject across all study sites, before and after ComBat harmonization. Median ODI values are shown in dots. B: Site-stratified ridge-line plots of whole-brain grey matter ODI before and after ComBat harmonization. Abbreviation: ODI = Orientation Dispersion Index, WL = West London, OX = Oxford, ED = Edinburgh, DB = Dublin, CB = Cambridge, ALFA = ALzheimer and FAmilies. |
| --- |


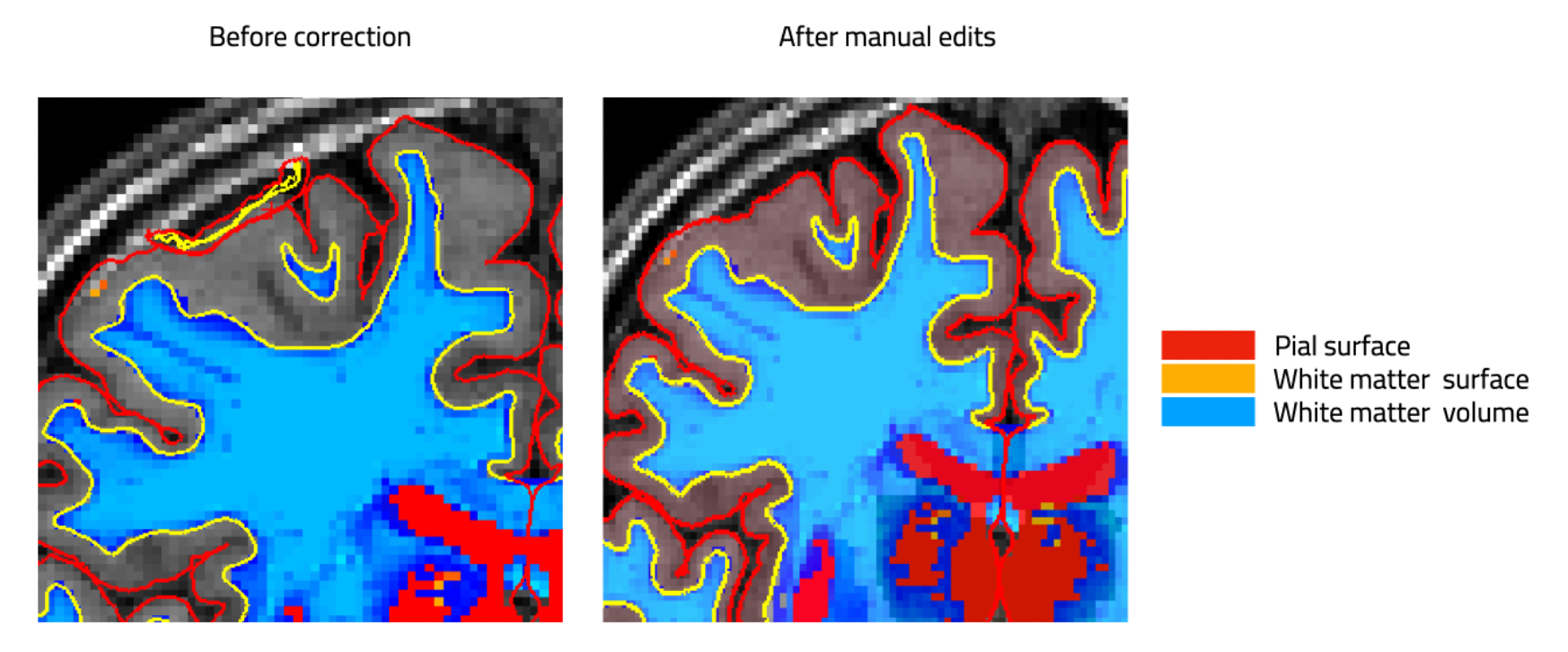


**Supplementary Fig 2.** Following visual inspection, manual edits were conservatively made to ensure correct definitions of pial and white matter surfaces.

# ComBat harmonization

## ComBat preserved biological variability of the data

The relationships between age and lobar ODI (averaged across both hemispheres) are presented in 2D density plots. There were strong linear and non-linear main effects of age with ODI across lobes (Age: *T _range_*= -19.5 - -4; Age^2^: *T* *_range_* = -10.9 - -5.8, p _FDR_ < 0.05) (Supplementary Fig. 3).

| 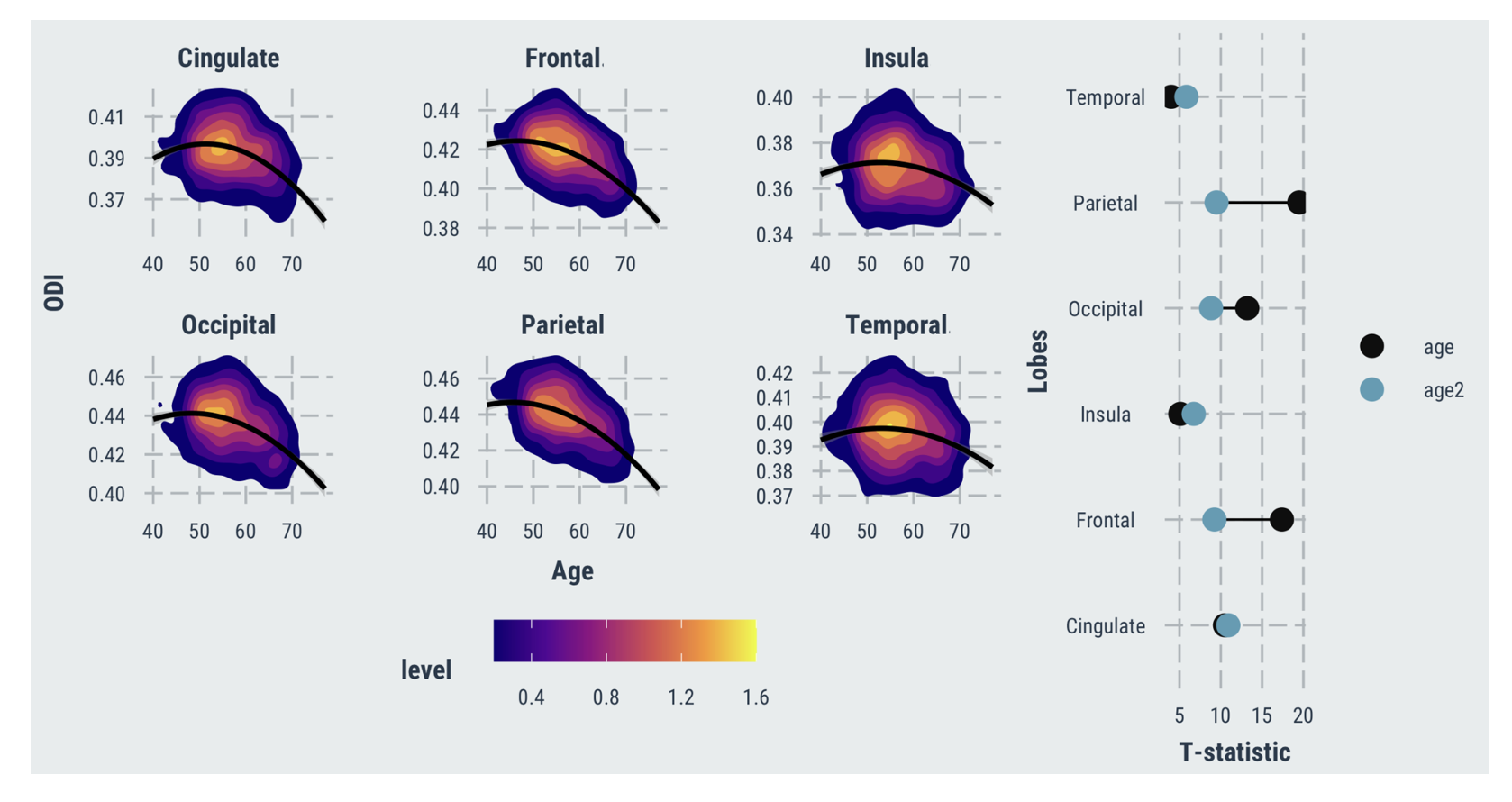  **Supplementary Fig 3.** Mains effect of age on ODI. Left: 2D density plots of age (x-axis) and lobar ODI (y-axis), averaged across both hemispheres. Right: T-statistics for the linear and non-linear age terms on lobar ODI. Abbreviations: ODI = Orientation Dispersion Index, *APOE* = Apolipoprotein E gene. |
| --- |

# Sensitivity analyses

## Excluding *APOE* ε2ε4 genotype

After the exclusion of ε2ε4 carriers, permutation models showed a widespread pattern of *APOE* ε4 × age interactions, reflecting steeper age dependent ODI changes in the ε4 carriers relative to non-carriers. Similarly, three-way interactions between APOE ε4 x age and education years were found, reflecting accelerated reductions of ODI in *APOE* ε4 carriers who have low levels of education (Supplementary Fig. 4 - 5).

| 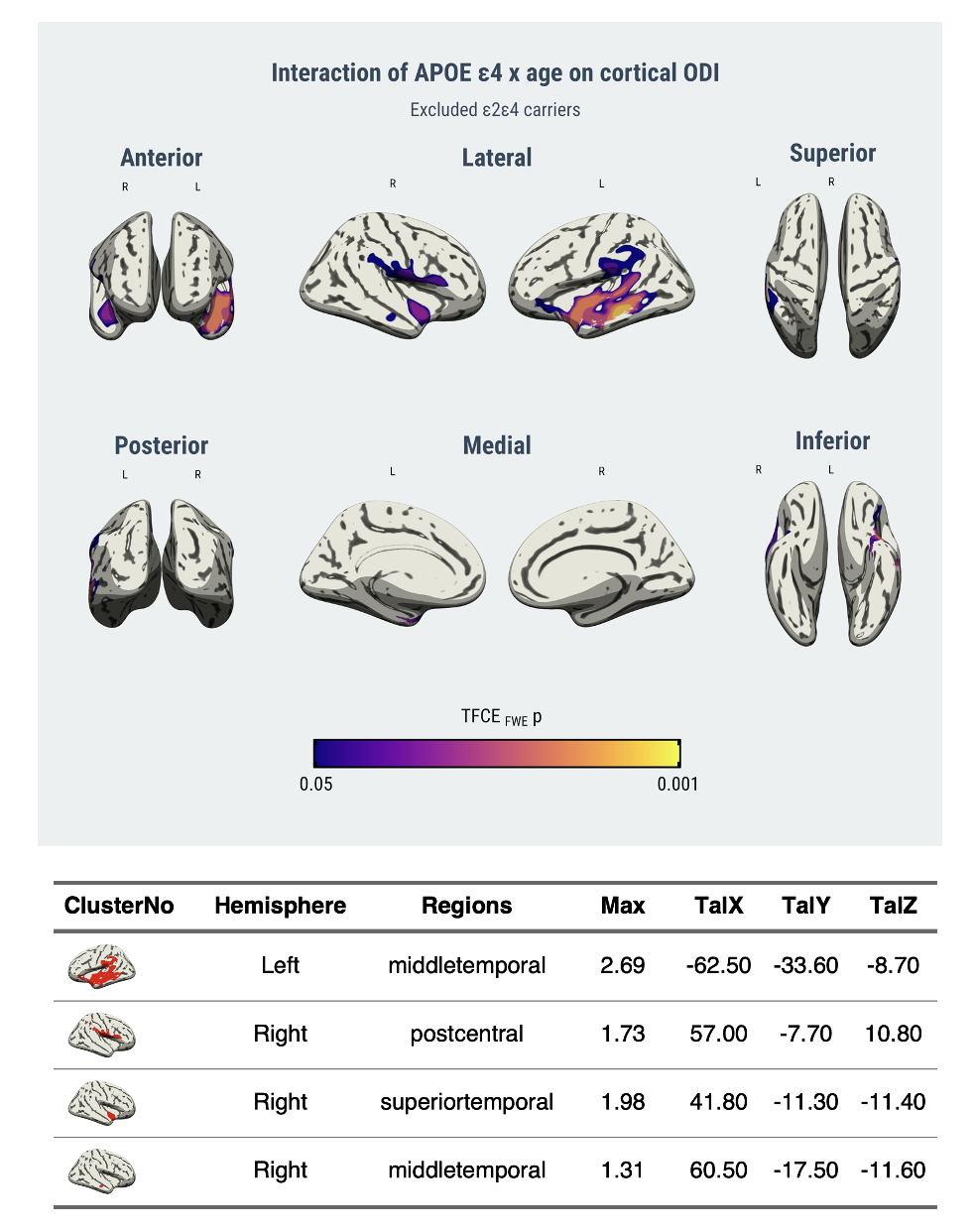  Supplementary Fig 4. Vertex-wise interactions between *APOE* ε4 and age on cortical ODI after excluding ε2ε4 carriers. Results were obtained using non-parametric permutation models, implemented using PALM on ComBat harmonized ODI surface maps, and significance was established with TFCE P _FWE_ < 0.05, adjusted for sex, years of formal education, and site. Abbreviations: APOE = Apolipoprotein E gene, TFCE = Threshold Free Cluster Enhancement, FWE = Family Wise Error, ODI = Orientation Dispersion Index, PALM = Permutation Analysis of Linear Models. |
| --- |

| 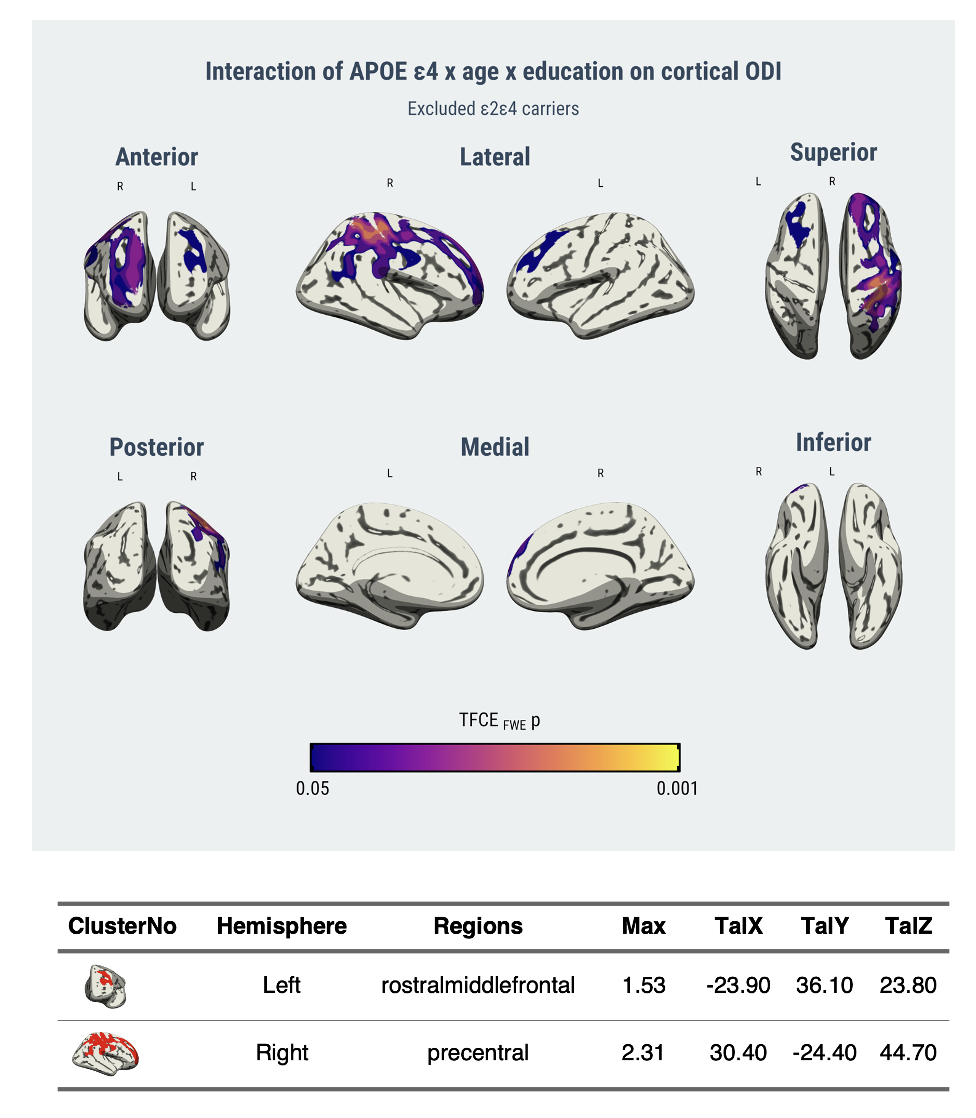 |
| --- |

Supplementary Fig 5. Vertex-wise interactions between *APOE* ε4 and age and education on cortical ODI after excluding ε2ε4 carriers. Results were obtained using non-parametric permutation models, implemented using PALM on ComBat harmonized ODI surface maps, and significance was established with TFCE P _FWE_ < 0.05, adjusted for sex, and site. Abbreviations: APOE = Apolipoprotein E gene, TFCE = Threshold Free Cluster Enhancement, FWE = Family Wise Error, ODI = Orientation Dispersion Index, PALM = Permutation Analysis of Linear Models.

## Controlling for mean cortical thickness

Given the mixed reports concerning the associations between *APOE* ε4 and cortical thickness changes, analyses were also repeated by mean cortical thickness as an additional covariate of no interest in our permutation models. In keeping with our primary findings, significant *APOE* ε4 x Age interactions were still found in an extensive pattern involving mainly the temporo-parietal cortices and frontal cortices (Supplementary Fig. 6).

| 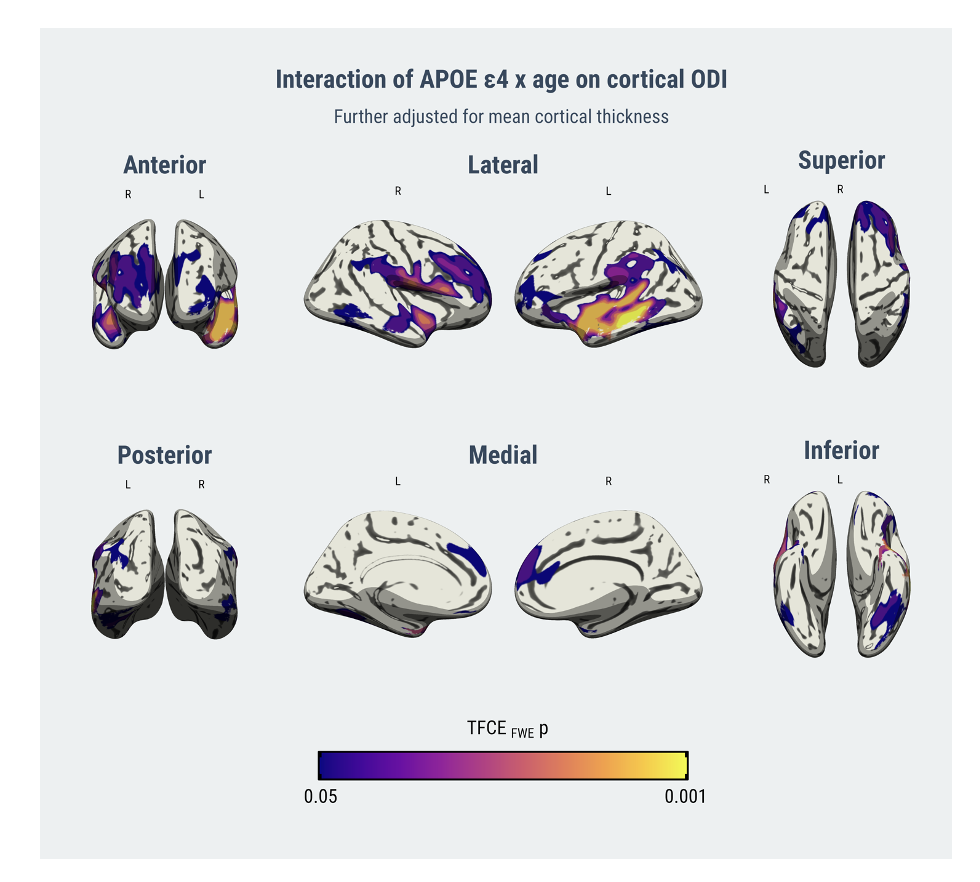  Supplementary Fig 6. Vertex-wise interactions between *APOE* ε4 and age on cortical ODI after adjusting for cortical thickness. Results were obtained using non-parametric permutation models, implemented using PALM on ComBat harmonized ODI surface maps, and significance was established with TFCE P _FWE_ < 0.05, adjusted for sex, and site, and cortical thickness. Abbreviations: APOE = Apolipoprotein E gene, TFCE = Threshold Free Cluster Enhancement, FWE = Family Wise Error, ODI = Orientation Dispersion Index, PALM = Permutation Analysis of Linear Models. |
| --- |
